# First-Principles Study of n*AlN/n*ScN Superlattices with High Dielectric Capacity for Energy Storage

**DOI:** 10.3390/nano12121966

**Published:** 2022-06-08

**Authors:** Wei-Chao Zhang, Hao Wu, Wei-Feng Sun, Zhen-Peng Zhang

**Affiliations:** 1 Key Laboratory of Engineering Dielectrics and Its Application, Ministry of Education, School of Electrical and Electronic Engineering, Harbin University of Science and Technology, Harbin 150080, China; weichaozhang@163.com (W.-C.Z.); 2020310226@stu.hrbust.edu.cn (H.W.); 2 School of Electrical and Electronic Engineering, Nanyang Technological University, Singapore 639798, Singapore; 3 Power Industry Quality Inspection and Test Center for Electric Equipment, China Electric Power Research Institute, Wuhan 430073, China; zhangzhenpeng@epri.sgcc.com.cn

**Keywords:** semiconductor superlattice, dielectric capacity, energy storage, first-principles calculation

## Abstract

As a paradigm of exploiting electronic-structure engineering on semiconductor superlattices to develop advanced dielectric film materials with high electrical energy storage, the n*AlN/n*ScN superlattices are systematically investigated by first-principles calculations of structural stability, band structure and dielectric polarizability. Electrical energy storage density is evaluated by dielectric permittivity under a high electric field approaching the uppermost critical value determined by a superlattice band gap, which hinges on the constituent layer thickness and crystallographic orientation of superlattices. It is demonstrated that the constituent layer thickness as indicated by larger n and superlattice orientations as in (111) crystallographic plane can be effectively exploited to modify dielectric permittivity and band gap, respectively, and thus promote energy density of electric capacitors. Simultaneously increasing the thicknesses of individual constituent layers maintains adequate band gaps while slightly reducing dielectric polarizability from electronic localization of valence band-edge in ScN constituent layers. The AlN/ScN superlattices oriented in the wurtzite (111) plane acquire higher dielectric energy density due to the significant improvement in electronic band gaps. The present study renders a framework for modifying the band gap and dielectric properties to acquire high energy storage in semiconductor superlattices.

## 1. Introduction

Today renewable sources are urgently developed and expected to dominate the future operation systems of electricity power. However, the inevitable intermittence bearing on renewable sources, such as solar and wind energies, challenges the continuous equilibrium required for temporarily storing electrical energy in adequately prolonged periods of time [1,2]. Even advanced batteries cannot respond sufficiently as fast to complement the promptly fluctuating energy sources [2]. In contrast, high-speed discharging electrostatic capacitors are uniquely preferable to efficiently fulfill the prompt complements in energy support systems [3]. Meanwhile, dielectric capacitors cannot be comprehensively applied to high-power energy storage until now due to the relatively low energy density of dielectric materials in electric discharging work.

In general, it is required for dielectric materials to achieve high energy storage density by increasing the maximum polarization intensity and breakdown field while persisting a low remnant polarization [4,5,6]. Comprehensive efforts have focused on pursuing antiferroelectric film materials with high energy storage density due to their double hysteresis loops of polarization-field characteristics, which can mostly approach the high-energy-density of 154 J/cm^3^, which is comparable with excellent electrochemical supercapacitors [7,8]. However, the energy storage performance of these antiferroelectric films requires a ferroelectric/antiferroelectric coexistence around the morphotropic boundary, which is intensively dependent on chemical composition and thermodynamic temperature [9,10,11]. It is also unfortunate for nonlinear dielectrics such as antiferroelectrics and relaxors that the inevitable energy dissipation in the charge/discharge cycle from hysteresis leads to low storage efficiency of recoverable energy. Moreover, for ferroelectric materials, it is difficult to approach a high energy density due to their substantial remnant polarization [12]. In comparison, linear dielectrics without remnant polarization and considerable energy loss it is only considered to acquire high energy density by promoting dielectric permittivity and breakdown field strength [13,14].

Recently arising linear dielectrics of III-V semiconductors in forms of solid solutions or superlattices, such as AlScN alloys or AlN/ScN superlattices, have attracted great focus for prospective energy storage due to their nonpolar phase in close proximity with ferroelectric states [15,16,17]. AlN is the most commonly used barrier material due to its largest band gap in the III-V group semiconductors, which is qualified for applying electric field as high strength as possible, and much promising for energy storage due to its chemical simplicity and low dielectric permittivity under high electric field. Recently observed ferroelectric states appearing in Al_1-x_Sc_x_N films with a substantial remnant polarization in contrast to pure AlN are actually polar or nonpolar but not in the ferroelectric phase [18,19]. The reactive magnetron sputtering method has been successfully applied to prepare Al_1__-x_Sc_x_N alloy film, which was expected to be improved for enhancing piezoelectric and ferroelectric responses [20,21]. From these works, it is worthwhile to investigate the AlN/ScN superlattices in a chemical component that is similar to the Al_1-x_Sc_x_N alloys as a representative of newly arising semiconductor film dielectrics with a preferable performance in terms of dielectric energy storage. The electronic band-edge characteristics of the semiconductor superlattices pivot on the quantum well confinement and band alignment of constituent layers, which accounts for the band gap and determines the electrical breakdown field of the electrical capacitor. Previous research lacks the proper consideration of the constituent layer thickness and crystallographic orientation of the AlN/ScN superlattices.

In the present study, we focus on the n*AlN/n*ScN superlattices oriented on the (001) or (111) crystallographic plane of a wurtzite structure, where n denotes the number of AlN or ScN monolayers in constituent layers of superlattices and indicates the constituent layer thickness. Their energy storage characteristics are studied by first-principle calculations of the band-structure and dielectric polarizability dependent on the electrical field and superlattice configurations to explore potential applications in high energy storage. Such artificial layered materials are generally fabricated by the epitaxial growth technology of controlling layer interface in an atom resolution, which provides great flexibility in optimizing electronic states and dielectric polarization by modifying the constituent-layer thickness and crystallographic orientation of superlattices. This also helps us to comprehend the underlying physics of high density and efficiency of energy storage in electrical capacitors.

## 2. Theoretical Methodology

The pseudopotential plane-wave method is used to carry out first-principle calculations of the crystal structure, electronic structure and polarizability by applying an electric field for (001) and (111) n*AlN/n*ScN superlattices, as implemented by CASTEP of Materials Studio 2020 (Accelrys Inc., Materials Studio version 2020.08, San Diego, CA, USA). The GGA-WC exchange-correlation function was adopted to perform geometry optimization and calculate the dielectric polarizability, while the HSE06 hybrid exchange-correlation function was specified to obtain accurate band structures [22]. The potential field of atomic cores bearing on the electrons is described by on-the-fly generated (OTFG) norm-conserving pseudopotential with the Koelling–Harmon treatment of relativistic effect [23]. Self-consistent field (SCF) iterations are implemented under convergence tolerance of 5 × 10^−7^ eV/atom in an FFT grid of 72 × 72 × 216, in which the Pulay scheme of charge density mixing in the magnitude of 0.5 is specified to relax the electrons [24,25]. The plane-wave basis-set with cut-off energy of 440.0 eV is modified by the basis-set finiteness correction [26]. Brillouin zone integration is realized by ***k*** point sampling on the Monkhorst-Pack 4 × 4 × 1 grid [27]. Crystal structures are geometrically optimized with the BFGS algorithm in delocalized internal coordinates under energy convergence of 5.0 × 10^−6^ eV/atom with a maximum of 0.02 eV/Å atomic force and 0.001 Å stress [28].

The internal electric fields are theoretically applied to the superlattice crystal structures along layer-plane normal (axis-*z*) to calculate hysteresis curves of electric polarization versus electric field strength (*P*-*E*), in which the geometry optimization for each electric field is performed to represent piezoelectric strain, and linear response formalism based on density-functional perturbation theory is enabled to calculate static dielectric permittivity under the direct-current internal electric fields [29]. According to the calculated band gaps, the intrinsic breakdown electric field is estimated empirically as the universal expression proposed by reference [30]. Cohesive energies in atom average are calculated by *E*_coh_ = n[*E*(Al) + 2*E*(N) + *E*(Sc)] − *E*(sup) where *E*(Al), *E*(N), *E*(Sc) and *E*(sup) represent total energies of Al, N and Sc isolated atoms, and superlattices.

## 3. Results and Discussion

### 3.1. Crystal Structure

**Figure 1:**
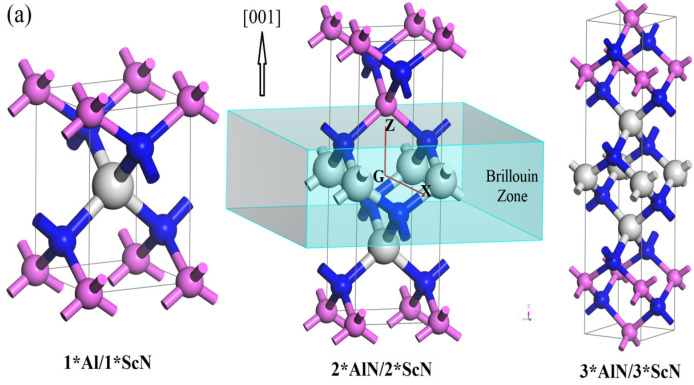

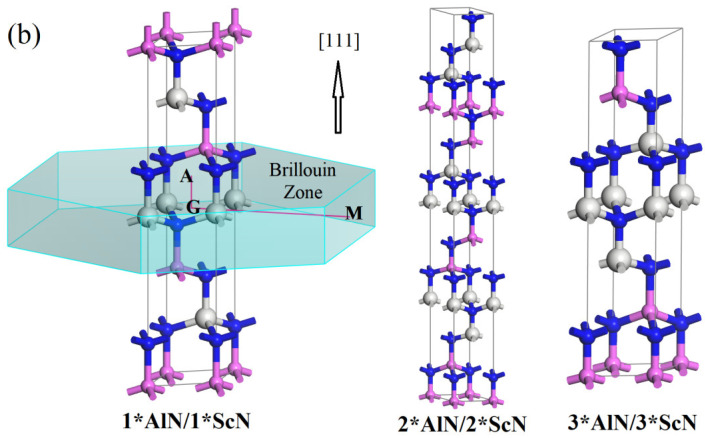
Crystal structures of n*AlN/n*ScN superlattices (*n* = 1, 2, 3) on (**a**) (001) and (**b**) (111) crystallographic faces of wurtzite structure, as indicated by layer-plane normal along (001) and (111) crystallographic orientations respectively, and the dispersion paths of electronic energy band through high symmetry points in the Brillouin zone are also shown. The gray, pink, and blue balls symbolize Sc, Al, and N bonding atoms, respectively.

Atomic configurations in crystal structures of (001) and (111) n*AlN/n*ScN superlattices (n = 1, 2, 3), as shown in Figure 1, have been energetically relaxed by geometry optimization without applying an internal electric field, indicating a diversity of space symmetries alternating with the adjustable superlattice parameters of n and crystallographic orientation. In addition, the space symmetry groups, lattice constants, the thicknesses of individual constituent layers, and cohesive energy per atom obtained are listed in Table 1. For the superlattice configurations, the ScN monolayer (double atomic layer) or the entire constituent layer for constructing superlattice structures is explicitly larger in thickness than the AlN monolayer or constituent layer, indicating that compressive and tensile strains of layer-plane exist in ScN and AlN layers respectively due to a lattice misfit. The thickness of the AlN constituent layer is strictly proportional to n while the ScN constituent layer becomes larger than n times the ScN monolayer thickness, implying that the Sc-N bonding elongation of the relaxing misfit strain along the layer-plane is normal when n increases, which also accounts for the higher cohesive energy per atoms of larger n than that of smaller ones.

The cohesive energy of the AlN/ScN superlattices approaches the highest and lowest values of 7.96 and 7.91 eV/atom for (001) 3*AlN/3*ScN and (111) 1*AlN/1*ScN superlattices, respectively, which are all remarkably higher than the III-chalcogenide covalent double-layers and TMD monolayers [31,32,33]. Bulk AlN and ScN are also calculated by identical first-principle schemes to obtain the cohesive energies of 7.66 and 8.38 eV/atom, which are slightly lower and higher, respectively, than these superlattices. It is an energetic manifestation of high structural stability that both (001) and (111) n*AlN/n*ScN superlattices can be feasibly achieved by matching the AlN and ScN monolayers through Sc-N or Al-N bonding strongly into a periodic layer structure. In comparison to the (001) superlattice orientation, the larger misfit in the (111) layer orientation accounts for the larger extension along the layer-plane normal with increasing constituent layer thickness and results in a lower cohesive energy per atoms.

### 3.2. Band Structure

**Figure 2:**
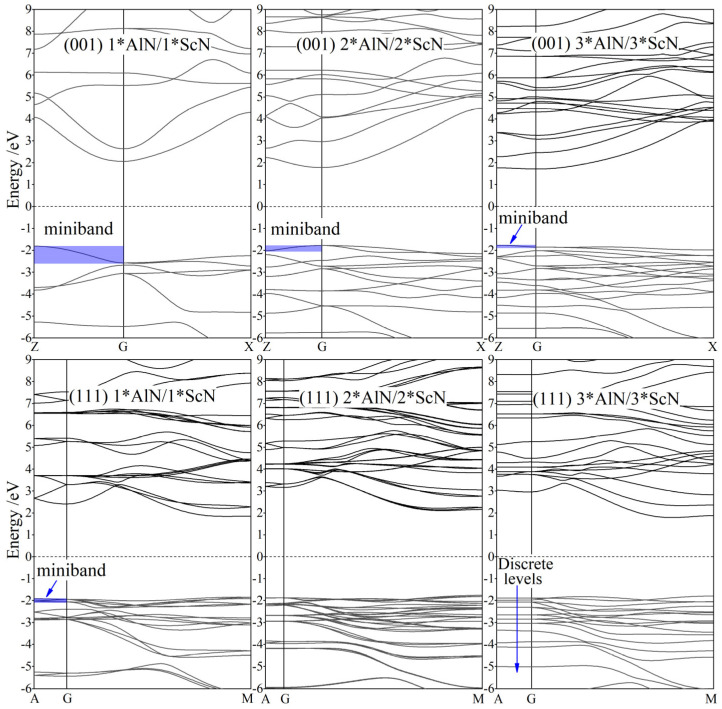
Band structures of the (001) and (111) n*AlN/n*ScN superlattices (*n* = 1,2,3) in the dispersion paths through high symmetry points in the Brillouin zone as indicated in Figure 1; the Fermi level (horizontal dash line) is referenced as energy zero.

Due to the in-layer quantum confinement and large lattice misfit between constituent layers in AlN/ScN superlattices, their band structures are quite different from AlN and ScN bulk materials, as shown in Figure 2. All of these superlattices present large electronic band gaps in the 3.5~4.5 eV range while persisting almost constantly without substantial dependence on constituent layer thickness (n), which is attributed to the simultaneous changing thickness of the individual constituent layer, almost fixing the quantum confinement levels or minibands of superlattices. In particular, for the higher values of n, a smaller dispersion along with the normal layer, as illustrated by the narrower minibands at the valence band-edge in Figure 2, indicates a more localized feature of valence electrons in response to the electric field perpendicular to superlattice layers, which manifests as a lower intensity of dielectric polarization.

The quantum confinement minibands of AlN/ScN superlattices are promptly narrowed down as the superlattice orientation is converted from the low symmetry (001) to high symmetry (111) crystallographic plane of a wurtzite-like structure due to the symmetry-induced degeneration of electronic energy levels. Even when n is raised to 3 for (111) orientation, the multiple electronic minibands with minimal energetic dispersion along with the superlattice’s normal layer, as in the *n* < 3 AlN/ScN superlattices, have shrunk into discrete energy levels due to the quantum well confinement of ScN constituent layer sandwiched by sufficiently wider AlN energy barriers. This results in notably larger band gaps of (111)-orientated superlattices than that of the (001) orientation, as shown in Table 1. For a more important consequence of a (111) orientation, the valence electrons are almost completely residing in ScN layers with a considerably lower dielectric polarizability than in the AlN layers, which are dominantly contributed by the uneasily polarized bonding of the valence band-edge electrons derived from the Sc-3*d* and N-2*p* orbitals. To this end, these electronic structure results elucidate why a higher electric polarization can be acquired whilst persisting with a large band gap by simultaneously increasing the AlN and ScN layer thicknesses.

### 3.3. Dielectric Polarization and Energy Density

**Figure 3:**
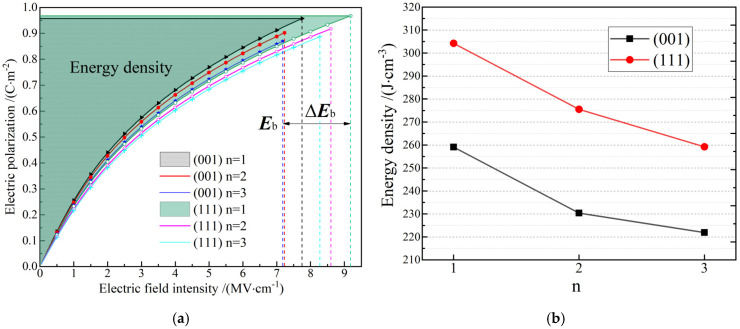
(**a**) *P*–*E* hysteresis curves where indicating breakdown field intensity by ***E***_b_ and energy density areas; (**b**) energy storage densities as an electric capacitor of (001) and (111) n*AlN/n*ScN superlattices (*n* = 1, 2, 3).

Dielectric polarization *P* under a high electric field has been evidently promoted by increasing the constituent layer thickness as indicated by a larger number of AlN or ScN monolayers in superlattice configurations, as shown in Figure 3a. The *P–E* relationship obtained from first-principle calculations at diverse points of the electric field intensity is fitted with the analytical functions of *E*(*P*) = *aP* + *bP*^3^ based on the Landau free-energy, whereby the energy storage densities are accurately evaluated by an analytical integral of *E*(*P*) as (*aP*_m_^2^/2) + (*bP*_m_^4^/4) where *P*_m_ denotes the electric polarization at intrinsic breakdown field, and no polarization arises under a zero external electric field, as shown in the results shown in Figure 3b. In contrast, in the (001) and (111) superlattice orientations, higher energy density can be acquired by the (111)-oriented superlattices, which is attributed to the significant improvement in band gap or intrinsic breakdown field strength for (111) AlN/ScN superlattices as listed in Table 1, whilst without considerable deficiency in dielectric polarization. Meanwhile, the increase of constituent layer thicknesses leads to higher dielectric polarizability under high electric fields for both the (001) and (111) AlN/ScN superlattices. The present first-principles calculations demonstrate that the n*AlN/n*ScN superlattices (*n* ≤ 3) are excellent nonlinear dielectrics of energy storage with the highest energy density approaching 304 J/cm^3^ by far exceeding the current supercapacitor materials realized in the experiments, as shown in Table 2.

It is clearly shown in Figure 1 that the majority of Al-N and Sc-N bonds are parallel to the layer-pane in (111) AlN/ScN superlattices which means all these polar bonds cannot contribute to dielectric polarization in response to the electric field perpendicular to layer-pane. In contrast, despite the diversion of an angle from the normal layer-plane in the (001) AlN/ScN superlattices, all of these ionic bonds of intrinsic dipoles are partially devoted to dielectric polarization under a normal electric field, as discriminated by the higher (001) electric polarizabilities than that of the (111) orientation. However, the (111) AlN/ScN superlattices possess remarkably larger band gaps and breakdown fields than the (001) AlN/ScN superlattices. Meanwhile, the larger out-of-plane tensile strain of the ScN constituent layer in the (111) AlN/ScN superlattices, as mentioned in Section 3.2, is another reason accounting for the larger band gaps and higher energy densities than the (001) AlN/ScN superlattices. It is thus flexible and preferable to exploit the superlattice configuration parameters, such as the constituent layer thickness and crystallographic orientation, to engineer band structures and dielectric responses of the AlN/ScN superlattices to suggest a feasible pathway for developing linear dielectrics for energy storage.

## 4. Conclusions

Employing a first-principle pseudopotential plane-wave method, the n*AlN/n*ScN superlattices with different constituent layer thicknesses and crystallographic orientations have been systematically studied by calculating the atomic structure, band structure and polarizability to elucidate their high energy density of the electrical capacity and predictable experimental feasibility. The large band gaps of the AlN/ScN superlattices can be retained, and the higher dielectric polarizabilities under a high electric field can be acquired by simultaneously increasing the numbers of AlN and ScN monolayers in individual constituent layers of superlattice configurations. The crystallographic orientation in the (111) plane will distinctively promote electronic band gaps while slightly decreasing the static dielectric response to the electric field normal to superlattice layers, which is respectively attributed to the increased absolute misfit of the superlattice layer and the in-plane orientations of major polar bonds. It is preferable to manipulate the superlattice configuration parameters to effectively adjust band structures and dielectric polarization of AlN/ScN superlattices. This study suggests a prospective routine of employing the highly controllable superlattice materials to steer electric polarizability and develop high energy density dielectrics.

## Figures and Tables

**Table 1:** The space symmetry group, lattice constant (*a*/*b*, *c*), thicknesses of AlN and ScN constituent layers (*h*_AlN_ and *h*_ScN_), and cohesive energy per atom (*E*_coh_), band gaps *E*_g_ and intrinsic breakdown field strength ***E***_b_ for the AlN/ScN superlattices.

Orientations	Superlattices	Space Groups	*a = b*/Å	*c*/Å	*h*_AlN_/Å	*h*_ScN_/Å	*E*_coh_/(eV/atom)	*E*_g_/eV	*E*_b_/(MV·cm^−1^)
(001)	1*AlN/1*ScN	P-4M2	3.2608	4.6528	2.0997	2.5531	7.9224	3.815	7.75
	2*AlN/2*ScN	PMM2	3.2525	9.3081	4.1715	5.1366	7.9542	3.559	7.23
	3*AlN/3*ScN	P-4M2	3.2507	13.9629	6.2454	7.7175	7.9642	3.535	7.18
(111)	1*AlN/1*ScN	R3M	3.2551	16.1767	2.4945	2.8978	7.9126	4.519	9.18
	2*AlN/2*ScN	R3M	3.2492	32.4172	4.9897	5.81603	7.9324	4.231	8.59
	3*AlN/3*ScN	P3M1	3.2487	16.2102	7.4845	8.7257	7.9380	4.072	8.27

**Table 2:** Energy densities of the AlN/ScN superlattices in comparison to the recently reported nonlinear dielectrics for energy storage capacitors, where the rGO and EDLC indicate reduced graphene oxide and electrochemical double-layer supercapacitor, respectively.

Material	Energy Density/J·cm^−^^3^	Method or Process
(001) 3*AlN/3*ScN superlattice	259	First-principles calculation
(111) 3*AlN/3*ScN superlattice	304	First-principles calculation
Nitrogen-Thiol-rGO Scrolls [34]	215	Nitrogen-doped thiol-functionalization
Pt(111)/Ti/SiO_2_/Si [35]	99.8	Solid-state reaction
rGO-based EDLC [36]	142	Hydrazine reduction

## Data Availability

Theoretical methods and results are available from the authors.
